# Supplementary material for: Salmonella enterica Serovar Typhimurium Isolates from Wild Birds in the United States Represent Distinct Lineages Defined by Bird Type
Source: Appl Environ Microbiol. 2022 Mar 22;88(6):e01979-21. doi: 10.1128/aem.01979-21 (PMC8939312; doi:10.1128/aem.01979-21)
Supplement: Supplemental file 1 — Fig. S1. Download aem.01979-21-s0001.pdf, PDF file, 0.3 MB [file aem.01979-21-s0001.pdf]

Supplementary Figure S1

```
LT2_sseL           1 VSDEALTLLFSAVENGDQNCIDLLCNLALRNDDLGHRVEKFLFDLFSGKRTGSSDIDKKI
passerine_sseL     1 VSDEALTLLFSAVENGDQNCIDLLCNLALRNDDLGHRVEKFLFDLFSGKRTGSSDIDKKI
larid_sseL         1 VSDEALTLLFSAVENGDQNCIDLLCNLALRNDDLGHRVEKFLFDLFSGKRTGSSDIDKKI
water_sseL         1 VSDEALTLLFSAVENGDQNCIDLLCNLALRNDDLGHRVEKFLFDLFSGKRTGSSDIDKKI
consensus          1 ************************************************************

LT2_sseL          61 NQACLVLHQIANNDITKDNTEWKKLHAPSRLLYMAGSATTDLSKKIGIAHKIMGDQFAQT
passerine_sseL    61 NQACLVLHQIANNDITKDNTEWKKLHAPSRLLYMAGSATTDLSKKIGIAHKIMGDQFAQT
larid_sseL        61 NQACLVLHQIANNDITKDNTEWKKLHAPSRLLYMAGSATTDLSKKIGIAHKIMGDQFAQT
water_sseL        61 NQACLVLHQIANNDITKDNTEWKKLHAPSRLLYMAGSATTDLSKKIGIAHKIMGDQFAQT
consensus         61 ************************************************************

LT2_sseL         121 DQEQVGVENLWCGARMLSSDELAAATQGLVQESPLLSVNYPIGLIHPTTKENI-------
passerine_sseL   121 DQEQVGVENLWCGARMLSSDELAAATQGLVQESPLLSVNYPIGLIHPTTKENI-------
larid_sseL       121 DQEQVGVENLWCGARMLSSDELAAATQGLVQESPLLSVNYPIGLIHPTTKENI-------
water_sseL       121 DQEQVGVENLWCGARMLSSDELAAATQGLVQESPLHLGKLSYWADSSYHQRKYIKHSAT*
consensus        121 ***********************************..................

LT2_sseL         174 --LSTQLLEKIAQSGL---SHNEVFLVNTGDHWLLCLFYKLAEKIKCLIFNTYYDLNENT
passerine_sseL   174 --LSTQLLEKIAQSGL---SHNEVFLVNTGDHWLLCLFYKLAEKIKCLIFNTYYDLNENT
larid_sseL       174 --LSTQLLEKIAQSGL---SHNEVFLVNTGDHWLLCLFYKLAEKIKCLIFNTYYDLNENT
water_sseL       180 KDCSIRIISQ*SLPGKYRRSLASLFIL*TCR------KNKMPYI*YLL*FK*KY*ARDYR
consensus        181   .*..........*.   *....*...*..........*.......*.*...*......

LT2_sseL         229 KQ-EIIEAAKIAGISESDE------VNFIEMNLQ---NNVPNGCGLFCYHTIQLLSN---
passerine_sseL   229 KQ-EIIEAAKIAGISESDE------VNFIEMNLQ---NNVPNGCGLFCYHTIQLLSN---
larid_sseL       229 KQ-EIIEAAKIAGISESDE------VNFIEMNLQ---NNVPNGCGLFCYHTIQLLSN---
water_sseL       228 SSKNCRHIRKR*G*FY*NEFTEQCTQRLWSILLPYNSTLIECRTKRSCYHTTRICGKFLN
consensus        241 .. ......*..*.....*      .......*.   ..........****......

LT2_sseL         276 --------AGQNDPATTLREFAENFLTLSVEEQALFNTQTRRQIYEYSLQ*
passerine_sseL   276 --------AGQNDPATTLREFAENFLTLSVEEQALFNTQTRRQIYEYSLQ*
larid_sseL       276 --------AGQNDPATTLR*FAENFLTLSVEEQALFNTQTRRQIYEYSLQ*
water_sseL       285 AFSRGTSTI*HPNPAANI*IQS------PV---------------------
consensus        301         .....**..............*....................
```

FIG S1 Comparison of SseL from different wild bird isolates. Multiple sequence alignment of SseL amino acid sequences from the larid, passerine, and water bird lineages. SseL from *S.* Typhimurium LT2 served as a reference.
